# Where do “chemical imbalance” beliefs come from? Evaluating the impact of different sources

**DOI:** 10.3389/fpsyg.2024.1469913

**Published:** 2025-01-08

**Authors:** Hans S. Schroder, Jordyn Tovey, Reni Forer, William Schultz, Elizabeth T. Kneeland, Jason S. Moser

**Affiliations:** ^1^Department of Psychiatry, University of Michigan Medical School, Ann Arbor, MI, United States; ^2^William Schultz Counseling, St. Paul, MN, United States; ^3^Amherst College, Amherst, MA, United States; ^4^Department of Psychology, Michigan State University, East Lansing, MI, United States

**Keywords:** chemical imbalance, etiological beliefs, depression, doctor-patient communication, beliefs

## Abstract

**Introduction:**

Although the etiology of depression is incredibly complex, the narrative that it is caused by a simple “chemical imbalance” persists in lay settings. We sought to understand where people are exposed to this explanation (i.e., the “source”), and the relative influence of each source.

**Methods:**

A total of 1,219 college students were asked where they had heard of the chemical imbalance explanation and how much they believed this to be true. Independent raters coded open-ended responses and we used self-report measures to capture chemical imbalance belief endorsement.

**Results:**

The most common sources of exposure to this explanation were the classroom, the Internet/media, other people (e.g., friends), and healthcare providers. In a regression analysis, only learning about the chemical imbalance explanation from healthcare providers uniquely predicted the adoption of the chemical imbalance belief. The correlation held even after controlling for depression symptoms, a family history of depression, and having had a diagnosis or treatment of mental health disorder (all of which also uniquely predicted chemical imbalance belief endorsement).

**Discussion:**

These results suggest that healthcare providers play an important role in the dissemination of the chemical imbalance message, which is an oversimplified, scientifically controversial, and potentially treatment-interfering narrative. Interventions directed at healthcare providers may help them engage with more accurate messages.

## Introduction

In July 2022, Joanna Moncrieff, psychiatrist and professor at the University of College London, and her colleagues published a review article demonstrating that depression was not reliably linked to the neurotransmitter serotonin (Moncrieff et al., 2022). The paper and related media coverage generated thousands of responses and divisive discussion. Leading psychiatrists claimed the article was “much ado about nothing” (Pies and Dawson, 2022), arguing that psychiatry has never seriously espoused a “chemical imbalance theory of depression.” However, the results were shocking to many non-professionals, who claimed they were duped by pharmaceutical advertising and their doctors. Days after the article was published, the radio station WBUR (Boston’s National Public Radio station) hosted a 1-h program with a psychiatrist answering phone calls from listeners who felt convinced their depression was the result of a chemical imbalance.

In its essence, the chemical imbalance explanation states that depression is the result of a chemical imbalance– most often portrayed as a “deficiency in serotonin.” The simple hypothesis that irregularities in one or a subset of monoamines such as serotonin, dopamine, or norepinephrine is responsible for depression has been rejected as having no scientific basis for decades (France et al., 2007; Hindmarch, 2001; Moncrieff et al., 2022), and yet its endorsement is widespread. The current study examines two questions: (1) *where* are people exposed to the chemical imbalance explanation? and (2) *how influential are these sources* on people’s actual beliefs about a chemical imbalance? Before we get to these questions, we review how this explanation rose to popularity in the first place and the psychological impacts of these explanations.

### Promotion of the chemical imbalance narrative by the pharmaceutical industry

Histories of psychiatry describe how different causal explanations have been more or less popular during different eras. Although the earliest physical explanations of melancholia date back to Hippocrates, it was not until the late 20th century that the specific “chemical imbalance” term came into prominence. According to some histories (Hengartner, 2021; Shorter, 2014; Whitaker, 2010), aggressive efforts by pharmaceutical companies for new medications specifically marketed for the new diagnosis of major depressive disorder in the 1980s and 1990s gave rise to the now-dominant biomedical language used today.

**Figure 1:**
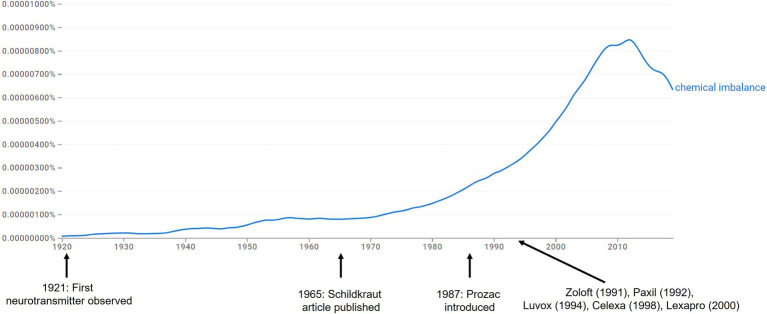
Google Ngram showing popularity of “chemical imbalance” phrase.

One way to understand the prevalence of a particular explanation is to calculate and examine its frequency in written text, which is made by possible with the Google Books Ngram Viewer (Michel et al., 2011). This tool uses the complete text corpus from Google Books, a massive library comprising millions of digital books, and evaluates the frequency of a given search term across time. Figure 1 displays a Google Ngram Viewer graph showing the frequency of the phrase “chemical imbalance” from 1920 to 2019. As illustrated, the publication of the Schildkraut (1965) article, one of the earliest formal articulations of a chemical imbalance-like hypothesis for affective disorders (although that particular article offered a quite nuanced hypothesis in this regard) was not associated with any great rise in popularity of the phrase. Several studies in the 1960s and 1970s examining the levels of serotonin, norepinephrine, and dopamine among individuals with and without depression failed to find evidence for the simple monoamine hypothesis (France et al., 2007; Harrington, 2019; Healy, 1997; Shorter, 2014) and was also unassociated with an increase in the phrase (Figure 1).

Rather than increasing in frequency alongside scientific discoveries, the popularity of the “chemical imbalance” term began its meteoric rise after the introduction of the selective serotonin reuptake inhibitors (SSRIs) in the mid-1980s. These products started with Prozac and extended to blockbuster medications including Lexapro, Celexa, Zoloft, and Paxil, all of which were approved for depression in the US between 1988 and 2002. Notably, policy changes in the late 1970s allowed for pharmaceutical companies to market directly to consumers (Harrington, 2019), and the introduction of these medications was accompanied by an onslaught of advertisements in medical journals, magazines, and TV commercials that portrayed depression as the result of a chemical imbalance (see Grow et al., 2006; Harrington, 2019; Leo and Lacasse, 2008), as well as large-scale public health campaigns promoting the biomedical model (Pescosolido et al., 2010). Together, these data suggest that the popularity of the phrase chemical imbalance had more to do with marketing strategies than scientific discoveries of true chemical imbalances.

### Psychological consequences of the chemical imbalance belief

Despite the lack of scientific credibility of the simple monoamine hypothesis, endorsement of the chemical imbalance belief abounds in many settings, particularly in the United States (Ang et al., 2022; France et al., 2007; Leo and Lacasse, 2008; Link et al., 1999).

Several reviews and meta-analyses have found that biogenetic messages and beliefs such as the chemical imbalance explanation have both benefits and costs (Kvaale et al., 2013; Lebowitz and Appelbaum, 2019). Although these beliefs may reduce blame (Kvaale et al., 2013), they have been tied to several detrimental consequences, including increased stigma toward people with mental health conditions (Kvaale et al., 2013), and internalization of poor recovery expectations (Kemp et al., 2014; Lebowitz et al., 2021; Schroder et al., 2020). Additionally, some studies suggest that providing chemical imbalance explanations also may influence preference for medication versus psychotherapy (Kemp et al., 2014), which is problematic as these two treatments offer largely equivalent effect sizes for most cases of depression (DeRubeis et al., 2005). These findings have spurred a call to action to reevaluate the emphasis on biogenetic factors when discussing depression and mental health problems more broadly (Deacon, 2013).

### Where have people been exposed to the chemical imbalance belief?

Most analyses of *where* individuals learn about the chemical imbalance explanation have focused on antidepressant advertisements (Grow et al., 2006) and related media coverage (Leo and Lacasse, 2008). Indeed, a study published 17 years ago found most college students had learned about the chemical imbalance explanation from direct-to-consumer advertisements (France et al., 2007). However, recent analyses have found that college textbooks offer simplistic chemical imbalance explanations of depression (Hunter, 2013) and that most students were exposed to the explanation in the classroom (Schroder et al., 2022), indicating a shift in how this explanation has been disseminated (i.e., from advertisements to curriculum). Healthcare providers offer another important source – a survey of social workers found that 90% used the chemical imbalance explanation with patients (Acker and Warner, 2020), and many patients report that they learned about the chemical imbalance explanation from their doctor.

### How influential are the different sources for chemical imbalance belief endorsement?

Our second question examined the *relative influence* of each source on the actual endorsement of the chemical imbalance belief. In other words, does learning about the chemical imbalance explanation from a healthcare provider carry more weight than learning about the theory on the Internet, or from a friend? Understanding which sources are most influential is paramount for initiatives aimed at correcting misconceptions of depression. Past research indicates a link between chemical imbalance belief and a personal history with depression and its treatment. For example, in a survey study of nearly 700 U.S. respondents recruited from an online marketing company, Park and Ahn (2013) found that chemical imbalance beliefs were related to personal and interpersonal experiences with depression, even more so than exposure to pharmaceutical advertising. Similarly, among a sample of 1,829 New Zealand adults who had been prescribed an antidepressant medication, Read et al. (2015) found that stronger chemical imbalance belief endorsement was associated with the perceived effectiveness of their medication. Among 279 patients in an intensive psychiatric treatment facility, there was a correlation between chemical imbalance beliefs and the number of previous psychiatric hospitalizations (Schroder et al., 2020). Finally, the chemical imbalance belief was more strongly endorsed among college students with a family history of depression, a mental health diagnosis, and having received mental health treatment (Schroder et al., 2022).

### The role of race and ethnicity

An additional consideration is how race and ethnicity play a role in the adoption of chemical imbalance beliefs. Previous research has found racial and ethnic differences in etiological beliefs about mental health, including depression (Jacobs et al., 2008). For example, an older study comparing White vs. Black respondents found that Black participants were less likely to adopt genetic or familial explanations of depression (Schnittker et al., 2000). Moreover, previous research has found that Black, Indigenous, and People of Color (BIPOC) face different experiences when seeking out formal treatment for mental health problems compared to White people, including increased stigma (Alvidrez et al., 2008). We are unaware of any studies evaluating the sources of different etiological explanations of depression in the context of race and ethnicity.

### The current study

This study was aimed to extend these previous studies in several ways. First, we included a very large sample of over 1,200 participants with wide-ranging experiences of depression and its treatment. Second, we evaluated several different sources of exposure to the chemical imbalance explanation of depression (e.g., classroom learning, healthcare providers, various forms of media), and examined their relative influence on actual endorsement of the belief using a regression analysis. Finally, we included several covariates in our analysis to control for other factors that have been shown to influence chemical imbalance belief, including depressive symptoms, family history, and history of contact with the medical system (diagnosis, medications, and psychotherapy) in order to reliably examine the strength of different sources of the explanation relative to the adoption of the belief.

## Method

### Participants

Participants (*N* = 1,990) were undergraduate students recruited from three universities in the United States during the Fall semester of 2022 and the Spring semester of 2023 for a larger project examining etiological beliefs about depression who completed the survey for partial course credit^1^. Of the participants who passed one attention check item to screen out inattentive responses (*N* = 1,755), a total of 1,279 (73%) had heard of the chemical imbalance explanation. This constituted the full sample for data analysis (*M*_age_ = 19 years, *SD* = 1.37, 73% female sex assigned at birth, 27% male sex assigned at birth; 69.7% White, 13.3% Asian/Asian *American, 6.2% Black, 3.4% Middle Eastern, 0.2% American Indian or Alaskan Native, 6.4% “Other”/Not listed; 13.2% identified as Hispanic/Latinx), although sample sizes differ slightly in the remaining analyses.

The Institutional Review Boards of the three universities each approved the study. The University of Michigan’s Health Sciences and Behavioral Sciences Institutional Review Board, which considered the ethical implications of the study, determined the survey to be exempt and had minimal harm for participants. The IRB protocol number was HUM00210977. Participants did not need to sign a consent form due to the exemption status of the study, but instead read a consent page explaining that continuing would indicate their consent to participate.

### Measures

#### Chemical imbalance beliefs

Chemical imbalance belief was assessed with a single item from the Reasons for Depression Scale (RFD; Thwaites et al., 2004), which read as follows: “I have depression because I have a chemical imbalance” (1 = Definitely not a reason), 4 (Definitely a reason). Participants who did not identify as being depressed were instructed to answer the question about depression in general.

#### Patient health questionnaire-8 (PHQ-8)

The PHQ-8 (Kroenke et al., 2009) consists of eight depression symptom items (e.g., “Little interest or pleasure in doing things”) and participants rated their experience on a 0 (Not at all) to 3 (Nearly Every Day) scale corresponding to the last 2 weeks. Reliability for the PHQ-8 was high in this study (*α* = 0.91).

#### Treatment and family history

Three questions (all Yes/No responses) asked about lifetime experience of attending a therapy session, being prescribed a medication, or receiving a diagnosis of major depressive disorder. One question asked about the presence of a family history of depression.

#### Chemical imbalance exposures

One question asked, “Have you ever heard about or seen depression being caused by a “chemical imbalance” or “imbalance of chemicals in the brain”?”. Those who indicated “yes” were then asked where they had heard about this explanation using a text box. Two independent raters (JT and RF) coded all open-ended responses. Following the coding scheme published by Schroder et al. (2022), the eight categories were as follows: School/Class, Internet/Media, Healthcare providers, Other People, Personal Experience, Definition, Unsure, and Uncategorizable. Up to three separate codes were allowable for each participant – for instance, the response “I’m not sure, my doctor, I think, and from psych class” would receive codes for Unsure, Healthcare Provider, and School/Class. Note that no participant used more than 3 categories. Categories could not be double counted (e.g., “psychology class” and “biology class” would only receive 1 code for School/Class). The two coders achieved excellent inter-rater reliability (*k* = 0.88) on the first codes and then met as a team (with HSS) to discuss the remaining discrepancies until an agreement was reached on all codes. Responses were then coded so that all participants received a score for each of the eight categories (e.g., School/Class, Healthcare Provider, etc.): a 1 if the category was present in their response text and a 0 if it was absent. Table 1 shows examples of each category and endorsement counts.

**Table 1:** Coded categories of open-ended responses.

Category	Number of mentions	Example responses
School/class	443	“Psychology class”
Internet/media	342	“Online,” “news articles”
Healthcare providers	200	“My therapist,” “doctor,” “my psychiatrist”
Definition	61	“…it has to do with neurotransmitters”
Other people	219	“A close friend of mine”
Personal experience	15	“Through my own mental health…journey”
Unsure	149	“I cannot trace back where I heard it from”
Uncategorizable	151	“Yeah”

Number of mentions exceeds sample size as up to three categories could be counted per participant.

### Data preparation and analysis

The primary analysis consisted of correlations between variables and a linear regression model predicting chemical imbalance beliefs. Independent variables included all eight categories of sources from the open-ended exposure question, sex, age, and previously published predictors of the chemical imbalance belief endorsement: family history of depression, past diagnosis of depression, medication history, therapy history, and depression symptoms. The dependent variable was the chemical imbalance belief item. Sample sizes differ slightly depending on the analysis.

## Results

### Chemical imbalance belief endorsement and depression-related experiences

The full scale of the chemical imbalance belief item was endorsed (range 1–4, *M* = 2.26, *SD* = 1.19) with 40% of the sample rating the item a 3 or 4 (“definitely a reason”). In terms of treatment contact, there was substantial variability within our convenience sample of college students; 19.9% (*N* = 272) of the sample reported having received a diagnosis of major depressive disorder, 44% (*N* = 601) indicated a family history of depression, 45.6% (*N* = 623) had attended at least one therapy appointment, and 29.2% (*N* = 399) had been prescribed a psychiatric medication.

### Correlations

Table 1 presents endorsement counts for the categorized sources of learning about the chemical imbalance explanation. As shown, most students had learned about this explanation from the classroom, Internet and other media, other people (e.g., friends), and healthcare providers. Table 2 shows bivariate correlations between all variables. Note that “healthcare providers” and “personal experience” were the only sources that positively related to chemical imbalance belief endorsement (*r*s = 0.36 and 0.11, respectively). Internet/media exposure was negatively correlated with the chemical imbalance belief (*r* = −0.07, *p* < 0.05). Females tended to endorse the chemical imbalance explanation more than males (*r* = 0.15, *p < 0*.01), replicating previous studies of sex differences (Schroder et al., 2020; Schroder et al., 2022).

**Table 2:** Correlations between study variables.

	1.	2.	3.	4.	5.	6.	7.	8.	9.	10.	11.	12.	13.	14.	15.
1. Age															
2. Female sex	−0.09**														
3. Chemical imbalance belief	0.05	0.15**													
4. School/class	−0.03	0.06*	0.01												
5. Internet/media	0.03	−0.01	−0.07*	−0.20**											
6. Healthcare providers	0.01	0.06*	0.36**	−0.09**	−0.14**										
7. Definition	0.01	−0.05	−0.01	−0.15**	−0.12**	−0.09**									
8. Other people	−0.01	0.01	−0.02	−0.19**	−0.03	−0.05	−0.08**								
9. Personal experience	0.04	0.001	0.11**	−0.04	−0.001	0.03	−0.03	−0.03							
10. Unsure	0.01	−0.03	−0.04	−0.14**	−0.02	−0.15**	−0.08**	−0.10**	0.01						
11. Uncategorizable	0.02	−0.02	0.004	−0.22**	−0.16**	−0.07*	−0.07*	−0.12**	−0.02	−0.10**					
12. PHQ-8	0.01	0.20**	0.37**	−0.02	−0.02	0.22**	−0.06*	0.02	0.08**	−0.08**	0.01				
13. Lifetime diagnosis of MDD	0.07**	0.13**	0.46**	−0.01	−0.09**	0.37**	−0.03	0.01	0.14**	−0.06*	−0.003	0.36**			
14. Family history of depression	0.03	0.13**	0.35**	0.03	−0.07*	0.22**	−0.02	0.03	0.06*	−0.05	0.06	0.29**	0.34**		
15. Lifetime therapy appointment	0.07*	0.16**	0.38**	−0.06*	−0.07*	0.34**	0.001	−0.02	0.09**	−0.04	0.01	0.26**	0.45**	0.33**	
16. Lifetime medication prescription	0.11**	0.08**	0.46**	0.01	−0.08**	0.41**	−0.04	−0.03	0.10**	−0.07*	−0.01	0.28**	0.55**	0.33**	0.57**

*N* = 1,219. Rows 4–11 are the sources of chemical imbalance explanation exposure (0 = did not indicate this source, 1 = did indicate this source). PHQ-8, Patient Health Questionnaire-8 (a measure of depression). Lifetime diagnosis, family history, therapy, and medication variables are coded as either 0 (no) or 1 (yes). *p < 0.05, **p < 0.01.

### Multiple linear regression analysis

Table 3 presents the results of the multiple linear regression analysis. The full model was a significant predictor of chemical imbalance belief endorsement, *F*(15, 1,218) = 42.70, *p* < 0.001, adjusted *R^2^ = 0*.23. In terms of individual predictors, as shown in Table 3, female sex at birth, depression symptoms, family history, diagnosis, and treatment were all unique significant predictors of the chemical imbalance belief, which replicates previous findings (Schroder et al., 2020; Schroder et al., 2022).

**Table 3:** Regression analysis predicting endorsement of the chemical imbalance belief (*N* = 1,219).

Predictor	*b*	SE	*B*	*t*	*p*	95% CI of *b*	
Age	0.01	0.02	0.01	0.28	0.78	−0.04	0.05
**Female sex**	**0.13**	**0.07**	**0.05**	**1.97**	**0.049**	**0.0003**	**0.25**
School/class	0.10	0.07	0.05	1.46	0.14	−0.04	0.24
Internet/media	0.03	0.07	0.01	0.47	0.64	−0.11	0.17
**Healthcare providers**	**0.47**	**0.09**	**0.14**	**5.07**	**<0.001**	**0.29**	**0.65**
Definition	0.23	0.14	0.04	1.60	0.11	−0.05	0.51
Other people	0.02	0.08	0.009	0.30	0.77	−0.13	0.18
Personal experience	0.40	0.26	0.04	1.56	0.12	−0.10	0.91
Unsure	0.13	0.09	0.04	1.40	0.16	−0.05	0.31
Uncategorizable	0.10	0.10	0.03	1.01	0.31	−0.09	0.29
**PHQ-8**	**0.03**	**0.01**	**0.16**	**5.21**	**<0.001**	**0.02**	**0.04**
**Lifetime diagnosis of MDD**	**0.50**	**0.09**	**0.17**	**5.76**	**<0.001**	**0.33**	**0.67**
**Family history of depression**	**0.29**	**0.06**	**0.12**	**4.58**	**<0.001**	**0.16**	**0.41**
**Lifetime therapy appointment**	**0.15**	**0.07**	**0.06**	**2.14**	**0.033**	**0.01**	**0.29**
**Lifetime medication prescription**	**0.47**	**0.08**	**0.18**	**5.67**	**<0.001**	**0.31**	**0.63**

After age and female sex, the next eight rows refer to exposure to the chemical imbalance explanation of depression and are coded 0 = absent, 1 = present. PHQ-8, Patient Health Questionniare-8 (Depression symptoms). The last four rows are also coded 0 = absent, 1 = present. MDD, Major Depressive Disorder. Bolded rows indicate *p* < 0.05.

Critically, the only information source that significantly predicted endorsement of the chemical imbalance belief in the regression was “healthcare providers” (*b* = 0.47, *t* = 5.07, *p* < 0.001). The direction of the effect suggests that participants who had learned about the explanation from their healthcare provider had significantly greater endorsement of the chemical imbalance belief. None of the other sources were significant predictors (*p*s > 0.10).

### Analysis of subsample of those who had been diagnosed with major depressive disorder

We re-ran the multiple regression analysis among just those who had been diagnosed with major depressive disorder (*N* = 267 available for the analysis); all predictors in the model were the same except for previous diagnosis of MDD. The results were virtually identical to those derived from the full sample; healthcare provider was the only significant source predictor of chemical imbalance beliefs (*b* = 0.36, *B* = 0.19, *t* = 2.62, *p* = 0.009). Depression symptom severity (*b* = 0.21, *B* = 0.13, *t* = 2.12, *p* = 0.035) and having received medication (*b* = 0.34, *B* = 0.14, *t* = 2.20, *p* = 0.029) were also predictive of the belief.

### Analysis of healthcare providers

Given that exposure from healthcare providers emerged as the only significant predictor in the adoption of the chemical imbalance belief, we examined more closely what participants considered as their “healthcare providers.” We re-examined the responses from the participants whose answers included a healthcare provider (*N* = 200) and then categorized the different types of providers. In order of frequency, these were as follows: “doctor” (*N* = 105 mentions), “therapist” (*N* = 70 mentions), “psychiatrist” (*N* = 32 mentions), “psychologist” (*N* = 10 mentions), “mental health professional” (*N* = 3 mentions), “medical professional” (*N* = 3 mentions), endocrinologist (*N* = 1 mention), and “hospital” (*N* = 1 mention). Note that some participants listed more than one healthcare professional, so the number of mentions exceeds the subsample size of 200. These results indicate that “doctor” and “therapist” were by far the most listed healthcare professionals that students indicated as espousing the chemical imbalance explanation. Although we cannot know definitively what these terms meant to students, it may be reasonable to assume that “doctor” referred to a non-specialized primary care physician, given that these providers play an increasingly outsized role in the administration of mental health services in the U.S. (see Olfson, 2016; Olfson et al., 2014).

### Analyses of ethnic/racial differences

To evaluate our sample in terms of race and ethnicity while maximizing statistical power, we categorized participants based on their responses on the Demographics form as White (*N* = 854) and participants who indicated categories other than “White” when completing the Race/Ethnicity form, which included the following categories: Asian or Asian American Black or African American, Native Hawaiian or Other Pacific Islander, Middle Eastern or North African, and “Other” on the Demographics page [hereafter: Black, Indigenous, and People of Color (BIPOC); *N* = 365].

An independent-samples *t*-test replicated previous research (Schnittker et al., 2000) that the chemical imbalance belief was more strongly endorsed among White participants (*M* = 2.36, *SD =* 1.21) compared to BIPOC participants [*M* = 2.02, *SD =* 1.13; *t*(774.53) = 4.88, *p* < 0.001, Cohen’s *d* = 0.29].

We then separately examined the primary regression analysis with source categories, PHQ-8, family history, medication and therapy history as the predictor variables and chemical imbalance belief endorsement as the dependent variable (i.e., Table 3) for White respondents and BIPOC respondents separately. The results were virtually identical to results from the full sample in each of these subsamples. Among participants identifying as White, exposure from healthcare providers was still a significant predictor of belief endorsement (*b* = 0.49, *B* = 0.15, *t* = 4.62, *p* < 0.001), although the “uncategorizable” source was also a significant predictor in this subsample as well (*b* = 0.24, *B* = 0.06, *t* = 2.02, *p* = 0.044). Depression symptoms, family history, medication history, and MDD diagnosis history were also all significant predictors. However, history of psychotherapy was *not* a significant predictor among the sample identifying as White (*b* = 0.03, *B* = 0.013, *t* = 0.37, *p* = 0.71). The healthcare providers category was the only significant source category predictor for BIPOC participants (*b* = 0.39, *B* = 0.11, *t* = 2.05, *p* = 0.041). Among BIPOC participants, depression symptoms, family history, therapy, and MDD diagnosis were significant predictors as well. However, medication history was *not* a significant predictor of chemical imbalance belief endorsement (*b* = −0.16, *B =* −0.05, *t =* −0.87, *p* = 0.39).

The results of the race/ethnicity analysis suggest that the overall, exposure from healthcare providers was still the most important predictor of chemical imbalance endorsement out of all the source categories. One difference between these subsamples was the predictive power of therapy vs. medication history in the endorsement of the belief: among participants identifying as BIPOC, having had a therapy appointment was a significant predictor of the belief (and medication history was *not*); the exact opposite pattern was true for White participants (medication history predicted this belief, but therapy history did not).

## Discussion

Here, we find that exposure to the chemical imbalance explanation of depression was prevalent, in that 73% of our large sample of students had heard of it. Most participants had learned about it from the classroom, Internet/media, other people, and healthcare providers. However, we find that only learning about chemical imbalance explanation from healthcare providers (primarily doctors and therapists) was uniquely and significantly related to the actual endorsement of this belief. These findings suggest that not all sources of information are equally impactful; rather, learning about this idea from professional healthcare authorities is especially convincing. We also find that personal and family history of the depression symptoms as well as mental health treatment also predicted chemical imbalance beliefs, replicating past findings (Schroder et al., 2022). Together, results suggest that contact with healthcare professionals is particularly important for the adoption of the chemical imbalance belief.

The direction of effects is telling. Exposure to the chemical imbalance theory from healthcare providers, having received a depression diagnosis, being prescribed medication, or engaging with therapy were all *positively* related to the adoption of the chemical imbalance belief. This suggests that participants’ providers may have shared/implied that a chemical imbalance is a reasonable, scientifically legitimate way of understanding depression. If, on the other hand, providers were routinely educating patients that this is an outdated, unsupported, and far too simplistic explanation of depression, or offering a more nuanced explanation of depression, there would be a negative correlation between exposure from healthcare providers and the adoption of these beliefs.

It is possible that both explicit messaging (telling a patient they have an imbalance) and implicit cues (e.g., prescriptions for antidepressants, doctor’s office, medical equipment) increase endorsement of medicalization conceptions including the chemical imbalance explanation in healthcare settings (see also Cohen and Hughes, 2012). Indeed, the number of psychiatric hospitalizations was positively correlated with the chemical imbalance belief in a separate study (Schroder et al., 2020). Note that we found that lifetime psychotherapy appointments were also uniquely predictive of this belief, suggesting that the influence on chemical imbalance endorsement is not limited to healthcare professionals who prescribe medication [we found that many of the healthcare providers listed were “therapists” (who presumably do not prescribe)]. However, the effect of having received medication was three times as predictive (*β* = 0.18) as the effect of having received therapy (*β* = 0.06), suggesting that explicitly *medical* intervention has a much stronger impact on the adoption of this belief. Moreover, the association between psychotherapy exposure and belief endorsement was limited to BIPOC participants (see more below).

### Implications for providers

As this belief has been linked to a variety of undesirable outcomes and attitudes including less hope for recovery and reduced sense of personal agency (Lebowitz and Appelbaum, 2019), these findings suggest that healthcare providers should explicitly educate patients about the nuanced, multifaceted nature of depression and counsel patients *against* the simple chemical imbalance explanation (see also Blease, 2014). We note that our analysis of *which specific* healthcare providers participants mentioned suggested that most of the providers were generic doctors and therapists (e.g., clinical social workers).

The American Psychiatric Association, American Psychological Association, American Counseling Association, and National Association of Social Workers Code of Ethics all require that medical professionals provide clients with informed consent (American Counseling Association [ACA], 2014; American Psychiatric Association [APA], 2013; American Psychological Association [APA], 2017; National Association of Social Workers [NASW], 2021). Informed consent includes a client’s right to accurate and comprehensive information related to their diagnosis, available treatments, prognosis, and possible adverse effects (Pouncey and Merz, 2019). Informed consent is ethically and professionally required for numerous reasons, including protecting the autonomy and self-determination of clients (Fisher and Oransky, 2008) in addition to promoting shared decision making in medical contexts (Zisman-Ilani et al., 2021).

Blease (2014) argued that robust informed consent also requires medical providers to be “well informed” and “up-to-date” (p. 225) regarding information related to medical care *as well as* awareness of common misunderstandings orbiting their areas of practice. When this obligation is applied to the diagnosis and treatment of depression, it follows that providers ought to proactively ensure that their clients understand that, while the brain is involved in all psychological experiences (including the experience of depression), there is no evidence that depression is *caused* by a “chemical imbalance” in their brain. Providers may instead state, “We do not yet understand exactly what causes depression, as it is very complicated. It is certainly not as simple as a deficiency in a single neurotransmitter.”

### Healthcare provider exposure

Understanding where healthcare providers learn about causal explanations of depression is an important next step. We are unaware of any published study that has examined this question, although one of our ongoing studies suggests that many medical doctors learn about biological explanations of depression in medical school. It is possible that pharmaceutical industry efforts including advertisements, direct-to-physician promotion of messages and medications, as well as sponsored educational activities (Vassilas and Matthews, 2006) all play a role in disseminating the chemical imbalance belief among medication providers. Parallel efforts from pharmaceutical companies in disseminating messages portraying pain as a chronic, uncontrollable disease which should be treated with “pain killers” contributed significantly to the opioid epidemic (Keefe, 2021). Refining a message that is scientifically supported, stigma-reducing, and empowering for individuals to seek out the help they need will be useful in alleviating the burden of the experience of depression.

### Influence of race and ethnicity

Finally, we found that overall results were very similar when we examined the correlations among White and BIPOC participants: in both subsamples, exposure to the chemical imbalance explanation from healthcare providers uniquely predicted belief endorsement. These findings suggest that healthcare providers have an important power in disseminating etiological beliefs regardless of race/ethnicity of the patient. Interestingly, however, whereas lifetime history of *medication* predicted this belief among White participants, lifetime history of *therapy* predicted this belief among BIPOC participants. These findings suggest that the influence of particular types of healthcare providers may be experienced differently among different racial/ethnic groups. Further research on the experiences and etiological takeaways of individuals from various backgrounds will be necessary to understand the meaning of this finding.

We should note that a significantly higher percentage of White participants reported a history of both therapy (55%) and medication (37%) compared to BIPOC participants (36 and 17%, respectively, *Χ^2^s >* 36, *p* < 0.001). These findings dovetail with a recent systematic review finding that BIPOC individuals are more likely to seek help from community leaders than mental health providers (Devonport et al., 2023), including religious leaders (Upenieks et al., 2023). Interestingly, a recent survey from 2019 to 2020 found that religious leaders themselves adopt a chemical imbalance belief about depression (Holleman and Chaves, 2023). Although religious leaders did not show up in our open-ended response boxes and thus was not a coded category, future research may explicitly probe the extent to which participants have been exposed to this and other etiological narratives about depression from a variety of other sources.

### Limitations

There are several limitations to our study that warrant attention and may guide future research. First, participants were drawn from a Western-centric, majority White, college student sample. This limits generalizability of our findings. We are additionally limited in terms of statistical power to offer fine-grained analyses of race and ethnicity. However, while we realize that BIPOC are not a homogenous group and contain many different cultural and historical backgrounds, our analyses of racial/ethnic differences suggests that the role of healthcare providers was important in the endorsement of the chemical imbalance belief regardless of our categorization of participants as White vs. BIPOC. Furthermore, our sample was heterogenous in depression symptoms, diagnosis, family history, medication and therapy experience. Second, our methodology relied on retrospective recall of where students were exposed to a particular message about depression. The validity of this recall is difficult to determine, especially in the US, where biogenetic messages are predominant in various formats (Lebowitz and Appelbaum, 2019); indeed, many participants replied “I do not know” to the prompt. Third, the correlational nature of our study precludes any causal inference about directionality. It is possible, for instance, that participants who hold strong biogenetic beliefs were more likely to seek out treatment for their depression, resulting in the observed correlations here. However, this is unlikely given that in our analysis of just those who had received a diagnosis (thus holding treatment-seeking as a constant), the healthcare provider exposure-belief endorsement correlation was still present. And even if that were the case, our findings converge with previous studies (Acker and Warner, 2020) suggesting that healthcare providers do disseminate chemical imbalance messaging. Together, our results suggest that interventions targeting healthcare providers and broader systems of medical communication may be necessary to dispel unhelpful and inaccurate explanations of depression.

## Data Availability

The raw data supporting the conclusions of this article will be made available by the authors, without undue reservation.

## Footnotes

^1^Some of these data have been reported elsewhere (Ahuvia et al., 2024; Schroder et al., 2024a; Schroder et al., 2024b). However, all of the present analyses are novel.
